# Advances in fusion level selection and surgical approaches for adolescent idiopathic scoliosis based on the Lenke classification system: a narrative review

**DOI:** 10.1186/s12893-025-03481-9

**Published:** 2026-01-05

**Authors:** Ruiyuan Chen, Yu Xi, Tianyi Wang, Aobo Wang, Ziqian Ma, Minghui Liang, Shuo Yuan, Lei Zang, Ning Fan

**Affiliations:** Department of Orthopedics, Beijing Chaoyang Hospital, Capital Medical University, 5 JingYuan Road, Shijingshan District, Beijing, 100043 China

**Keywords:** Adolescent idiopathic scoliosis, Lenke classification system, Spinal fusion, Surgical planning, Selective fusion, Surgical approach, Upper instrumented vertebrae, Lower instrumented vertebrae

## Abstract

**Background:**

Adolescent idiopathic scoliosis (AIS) is a complex three-dimensional spinal deformity, frequently requiring fusion surgery. An optimal fusion surgical strategy can not only achieve effective correction but also reduce the incidence of postoperative complications. Recently, several researchers have refined and expanded AIS fusion surgical strategies based on the Lenke classification system, which is the current international standard for AIS. Therefore, this study aims to review the advances in fusion level selection and surgical approaches for AIS based on this classification.

**Methods:**

Databases such as PubMed, Embase, Web of Science, Scopus, Cochrane Database, China National Knowledge Infrastructure, Wanfang Database, and China Biomedical Literature Database were queried for articles using the keywords “adolescent idiopathic scoliosis”, “fusion surgery”, “Lenke classification system”, “Lenke 1”, “Lenke 2”, “Lenke 3”, “Lenke 4”, “Lenke 5” and “Lenke 6”.

**Results:**

Over the past decade, fusion surgical guidelines based on the Lenke classification have been refined, with new strategies emerging. We summarize the latest AIS fusion surgical strategies with recent research results. However, the fusion strategy based on the Lenke classification system has undergone no revolutionary changes. The selection of surgical designs for certain subtypes remains controversial.

**Conclusion:**

The fusion surgical strategy based on the Lenke classification system remains the standard for AIS surgical treatment. With the advancement of surgical technologies, further optimization of surgical strategies and the development of three-dimensional classification systems are potential future directions.

**Supplementary Information:**

The online version contains supplementary material available at 10.1186/s12893-025-03481-9.

## Introduction

Adolescent idiopathic scoliosis (AIS) is a complex three-dimensional (3D) spinal deformity of unknown etiology that impairs the physical and psychological well-being as well as quality of life of adolescent patients. Treatment modalities for AIS involve observation, bracing, and surgical intervention. Surgery is considered for skeletally immature patients with progressive curves greater than 40° and skeletally mature patients with progressive curves greater than 45° to 50° [1]. The surgical intervention aims to correct the scoliotic deformity, attain spinal stability, and preserve bodily balance. An optimal strategy for spinal fusion, including fusion level determination and upper and lower instrumented vertebrae selection, not only ensures effective deformity correction but also reduces postoperative complications incidence. However, the development of fusion surgical strategies for AIS has long been a focal point of debate.

The establishment of spinal scoliosis classification systems holds pivotal clinical significance for preoperative AIS assessment and fusion surgical strategy development. Prominent classification systems include the King, Lenke, and PUMC classifications. The King classification was the first to propose fusion strategies based on Harrington instrumentation. However, spinal correction has evolved from one-dimensional to 3D approaches with continuous advancements in orthotic devices and surgical techniques. Consequently, its fusion strategy has become inadequate for third-generation orthotic systems, such as Cotrel–Dubousset instrumentation, and pedicle screw and rod fixation system. In 2001, Lenke et al. [2] introduced the Lenke classification system, which has become the internationally accepted standard for AIS classification in recent years. Building upon this classification system, Lenke et al. [1, 3] further developed AIS fusion surgical guidelines in 2007, encompassing selective fusion, surgical approach, and upper and lower instrumented vertebrae selection. Further, several scholars have continuously refined and enriched AIS fusion strategies in recent years within the framework of the Lenke classification system. Therefore, this study aimed to provide a comprehensive review of the research advances in fusion level selection and surgical approaches for AIS based on the Lenke classification system.

## Methods

**Fig. 1:**
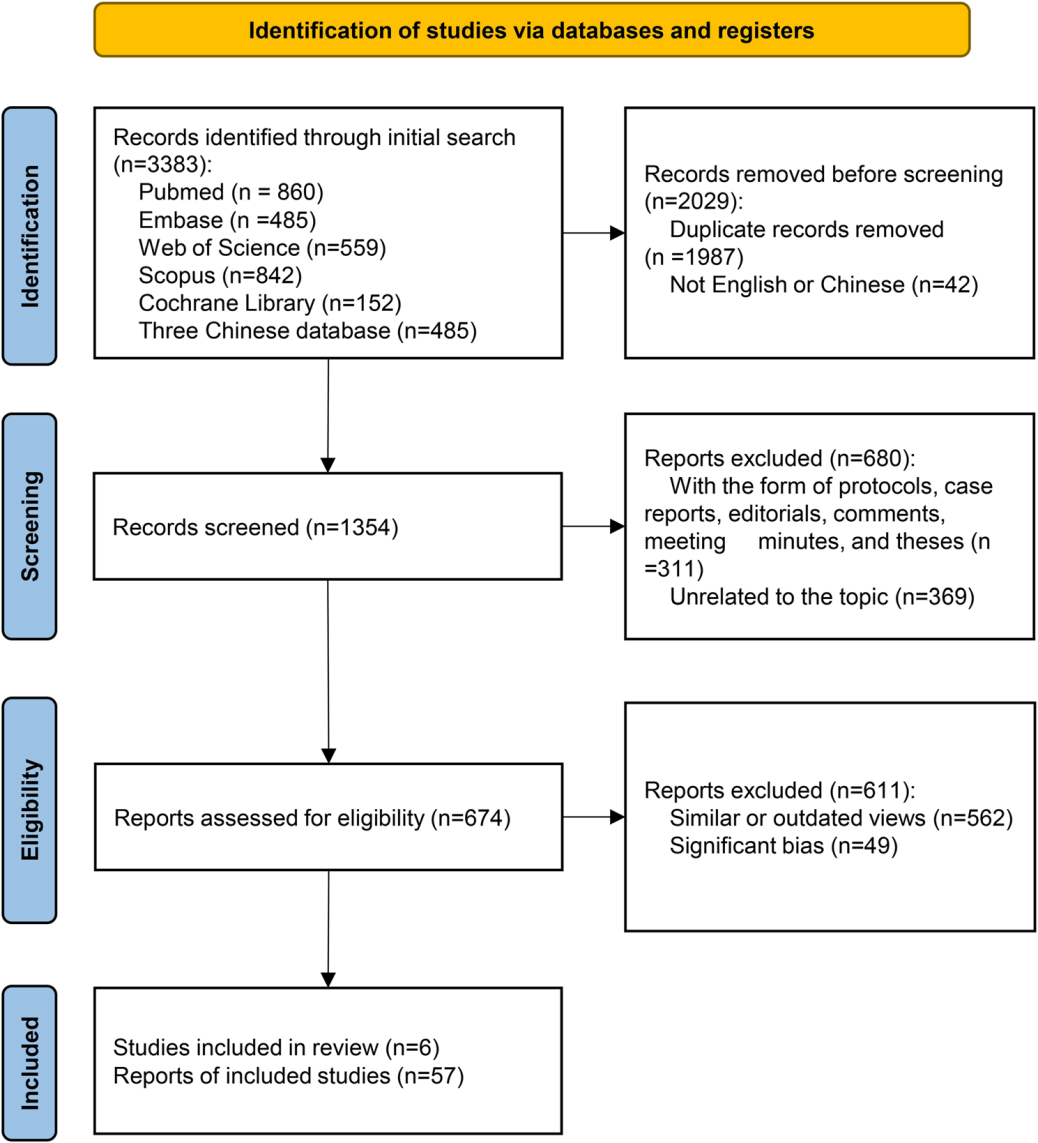
Flow diagram of this review

This is a narrative review. Databases such as PubMed, Embase, Web of Science, Scopus, Cochrane Database, China National Knowledge Infrastructure, Wanfang Database, and China Biomedical Literature Database were queried for articles using the keywords “adolescent idiopathic scoliosis”, “fusion surgery”, “Lenke classification system”, “Lenke 1”, “Lenke 2”, “Lenke 3”, “Lenke 4”, “Lenke 5” and “Lenke 6” that were published between 2001 and 2025. As in all narrative reviews, a selection bias cannot be excluded. Absence of linguistic restrictions further widened the scope of inquiry. To ensure a comprehensive literature search, two reviewers (RC and YX) independently screened prior articles and manually checked the reference lists of relevant publications to identify studies meeting the eligibility criteria. After duplicate records were removed using EndNote (Clarivate), titles and abstracts were screened to assess potential relevance. Study protocols, case reports, editorials, commentaries, and these were excluded. The remaining studies were subsequently assessed in full text, and those failing to meet the inclusion criteria were excluded. Any disagreements were resolved by consensus, with arbitration by a third reviewer (LZ) when required. Fig. 1.

## Results

### Lenke type 1

Lenke Type 1 is characterized by a main thoracic curve (MTC) that is structural, whereas the proximal thoracic curve (PTC) and the thoracolumbar/lumbar curve (TL/ LC) are nonstructural. Based on the lumbar spine modifier, Lenke Type 1 is further subdivided into 1 A, 1B, and 1 C subtypes. Surgical intervention for this type may involve selective thoracic fusion (STF) via either an anterior or posterior approach. Upper instrumented vertebra (UIV) selection must consider the Cobb angle and flexibility of PTC, as well as the overall shoulder balance. Lower instrumented vertebra (LIV) selection should reference either the stable vertebra (SV) or the touching vertebra (TV), as this selection is closely associated with the occurrence of distal adding-on phenomena postoperatively. SV is the most cephalad vertebra that is bisected by the central sacral vertical line (CSVL). TV is the most cephalad vertebra contacted by the CSVL and can be classified as substantially touched vertebra (STV) or non-STV (nSTV) based on whether the CSVL falls within or outside the pedicle margins of the contacted vertebra.

#### STF

Lenke Types 1 and 2 both consist of a structural MTC and a nonstructural TL/ LC, enabling a combined discussion. According to Lenke’s early perspective, the MTC in Lenke Types 1 and 2 is considered structural, whereas the TL/LC is nonstructural and highly flexible, indicating that STF would be feasible. However, Newton et al. [4], in 2003, revealed that only 68% of patients with Type 1 C underwent STF. A prospective study by Crawforad et al. [5] in 2013 reported an even lower rate of 49%. To investigate the underlying reasons, Boniello et al. [6] compared STF with non-STF procedures in 2015 and revealed that surgeons would prefer non-STF when the preoperative apical vertebral translation (AVT) of the TL/LC was greater. This preference is based on studies indicating that patients with Lenke Types 1 C and 2 C who undergo STF demonstrate considerable risks of postoperative complications, with reported incidences of coronal imbalance ranging from 20% to 38%, lumbar decompensation around 9%, and the adding-on phenomenon as high as 25% [7, 8].

To this end, Lenke et al. [9] proposed that STF for Lenke Types 1 C and 2 C should be performed under the following conditions: (1) MTC > TL/LC, with TL/LC of < 25° and/or junctional thoracolumbar kyphosis of ≥ 20° between T10 and L2 on side bending; and (2) the ratio criteria of MTC: TL/L Cobb magnitude, AVT, and apical vertebral rotation (AVR) must be in the range of 1.2 or greater. Moreover, Kaya et al. [10] indicated that STF provides satisfactory clinical and radiological spontaneous lumbar curve correction in Lenke Types 1 C and 2 C curves, whenever lumbar flexibility on the preoperative bending x-ray is greater than 70% and AVR is equal or less than grade 2. Furthermore, adjunctive bracing may be considered to prevent complications, such as coronal imbalance and decompensation of the lumbar curve after STF. However, research on the efficacy of bracing after STF is currently limited, and further studies are warranted to validate its feasibility and effectiveness.

In summary, patients with Lenke Types 1 A, 1B, 2 A, and 2B are suitable candidates for STF. However, STF should only be considered for patients with Lenke Type 1 C and 2 C if certain criteria are met. Otherwise, a higher risk of postoperative complications, such as coronal imbalance and lumbar curve decompensation, is possible.

#### Selection of surgical approach

Lenke et al. [1] have indicated that both anterior spinal fusion (ASF) and posterior spinal fusion (PSF) can be used for the correction of Lenke Type 1. However, most surgeons prefer PSF, which provides a better MTC correction rate and spontaneous TL/LC correction rate, although PSF involves more fused segments compared with ASF [11, 12]. On the contrary, ASF requires a transthoracic approach, which significantly increases the complexity of the procedure. Further, Newton et al. [12] indicate that open ASF is prone to pulmonary function impairment, requiring careful consideration for patients with AIS with compromised preoperative pulmonary function. While thoracoscopy-assisted ASF has exhibited satisfactory surgical outcomes, it is associated with longer operative times, higher complication rates, increased surgical complexity, and less effective deformity correction [12, 13]. Therefore, current evidence indicates that PSF is the preferred surgical approach for Lenke Type 1. Nevertheless, anterior release remains an important consideration in specific situations, such as very large and stiff coronal curves, pronounced lumbar lordosis, or in skeletally immature patients, where it can enhance curve flexibility, facilitate correction, and reduce the risk of crankshaft phenomenon [14, 15]. Thus, individualized assessment of curve rigidity and skeletal maturity is essential when determining the surgical approach.

#### Selection of UIV

UIV selection is strongly associated with the occurrence of postoperative shoulder imbalance. Depending on the preoperative shoulder imbalance and the flexibility of the PTC, a higher UIV may be selected to directly correct the shoulder imbalance through surgery, or a lower UIV may be chosen for spontaneous compensation of shoulder imbalance via the PTC. Based on the characteristics of PTC of Lenke Type 1, which is a nonstructural curve with good flexibility and spontaneous compensatory ability, Lenke et al. [3] indicated that the UIV selection should primarily depend on the preoperative balance of both shoulders and proposed the following principles for UIV selection: (1) select T2 as the UIV when the preoperative left shoulder is high; (2) select T3 or T4 as the UIV when the preoperative shoulders are level; (3) select T4 or T5 as the UIV when the preoperative right shoulder is high. However, a study by Chan et al. [16] revealed that when PTC ≥ 15° on side bending radiographs, its flexibility and spontaneous compensatory ability are similar to those of structural PTC of Lenke Type 2 (PTC ≥ 25° on side bending radiographs), indicating that fusion should be considered for PTC at this point.

Furthermore, some researchers have proposed different perspectives on UIV selection. Brooks et al. [17] revealed that selecting T4 as the UIV caused better shoulder balance postoperatively than T2 or T3, regardless of which shoulder was elevated preoperatively. Therefore, reducing the number of fused segments of the PTC to enable spontaneous compensation may be beneficial when the PTC exhibits good flexibility, thereby improving postoperative shoulder imbalance. In 2020, Munakata et al. [18] innovatively proposed a new UIV selection method that utilized the modified Shinshu line (MSL) to establish the selected UIV as the MSL vertebra (MSLV) by referring to the Shinshu line (a line connecting the centers of the concave-side pedicles of the UIV and LIV on preoperative radiographs) proposed by Oba et al. [19]. The MSL is a line connecting the center of the C7 spinous process and the center of the spinous process of the LIV. MSLV is the vertebral body with which MSL first contacts proximally. Munakata et al. [18] demonstrated selecting the MSLV as UIV achieved superior trunk and shoulder balance without compromising the main thoracic correction rate or overall sagittal alignment.

Besides, the MTC correction rate is also an important factor affecting postoperative shoulder imbalance. Excessive correction of the MTC, particularly when not accompanied by adequate adjustment of the PTC, is strongly associated with postoperative shoulder imbalance [20, 21]. The surgical strategy should focus not only on UIV selection but also on the balance of curve correction.

#### Selection of LIV

Lenke Types 1 and 2 both consist of structural MTC and nonstructural TL/LC, sharing similar principles for LIV selection. Lenke et al. [1, 3] have proposed the following criteria for LIV selection. For Types A and B, the recommended LIV is 1–2 vertebrae below the SV (SV-1 or SV-2), congruent with Suk et al.’s principle for LIV selection in single thoracic curves [22]. In contrast, for Type C, the lower end vertebra (LEV) may be selected as the LIV in anterior surgical approaches, whereas the SV (typically T11, T12, or L1) is preferred for posterior surgeries [1]. In 2020, Lenke et al. [23] further proposed the TV rule, recommending the TV as the LIV for patients with AIS with Lenke Types 1 and 2. A 2022 meta-analysis by Ifthekar et al. [24] compared the efficacy of selecting SV versus TV as the LIV, revealing no significant differences in scoliosis correction rates, patient satisfaction, or complication rates. However, selecting TV as the LIV reduced the number of fused segments. Consequently, TV is recommended based on existing studies.

LIV selection is critically associated with the occurrence and progression of postoperative distal adding-on. Surgeons strive to preserve as many mobile segments as possible while minimizing the incidence of adding-on. A meta-analysis conducted by Lenke et al. [25] in 2020 indicated that selecting STV/(nSTV + 1) as the LIV for patients with Type 1 A/2A results in the lowest incidence of distal adding-on. However, Qin et al. [26] demonstrated that selecting STV-1 as the LIV does not increase the incidence of adding-on when the Risser grade is higher, the thoracic curve is shorter, and the vertebral rotation and displacement at the vertebra immediately above the STV (STV-1) are minimal. Based on these results, they proposed the STV-1 rule for Type 1 A/2A. In addition to LIV selection, other risk factors for adding-on occurrences include younger age, lower Risser grade, larger preoperative Cobb angle in the main curve, thoracolumbar sagittal flexibility, and preoperative coronal and sagittal imbalance [27, 28]. Recently, Guo et al. [29] reported four best predictor variables for adding-on occurrences, including the number of vertebrae in the MTC, apical vertebra translation, LIV rotation, and Risser sign. Therefore, surgeons should consider appropriately extending the fused segments distally to reduce the adding-on incidence when these risk factors are present.

### Lenke type 2

Lenke Type 2 is characterized by double thoracic curves, with the MTC serving as the structural primary curve and the PTC as the structural secondary curve, whereas the TL/LC is nonstructural. It is further classified based on the lumbar spine modifier into Types 2 A, 2B, and 2 C. Surgical intervention for Lenke Type 2 may involve posterior approach fusion of the double thoracic curves. UIV selection should consider the balance of the shoulders. Further, LIV selection follows the same principles as for Lenke Type 1, requiring reference to either the SV or the TV. Notably, Lenke Type 2 curves demonstrate a significantly higher risk for a postoperative thoracic hypokyphosis [30].

#### UIV selection

Lenke et al. [1, 3] proposed that UIV selection should be guided by the preoperative bilateral shoulder balance as follows: (1) T2 is recommended when the preoperative left shoulder is high; (2) select T2 or T3 as the UIV when the preoperative shoulders are level; (3) T2 or T3 is advised when the preoperative right shoulder is high; (4) T4 or T5 may be considered in the rare cases where the PTC fusion is not necessary. However, this approach carries the risk that the left shoulder may not elevate postoperatively. Li et al. [31] involved 25 patients with UIV at T1 or T2 and demonstrated that 21 patients achieved shoulder balance postoperatively, with only 4 experiencing mild shoulder imbalance. In 2017, Jiang et al. [32] investigated the correlation between UIV and cervical tilt in a cohort of 30 patients with Lenke Type 2 presenting with right-elevated shoulder. The patients were divided into groups A (UIV at T1 or T2) and B (UIV at T3 or below). Both groups achieved satisfactory shoulder balance postoperatively, but group B demonstrated a significant increase in cervical tilt compared to preoperative measurements. Consequently, they recommended that for patients with Lenke Type 2 with right-elevated shoulder, a full fusion of proximal thoracic should be performed with the UIV selected as T1 or T2 to mitigate the risk of postoperative aggravation of the cervical tilt. In 2022, Munakata et al. [33] proposed an innovative approach by selecting the MSLV as UIV for patients with Lenke Type 2. This design significantly reduced the incidence of postoperative shoulder imbalance. Based on these results, we indicate selecting T1 or T2 as the UIV according to shoulder balance. However, careful deliberation is advised when selecting T3, as it may exacerbate cervical tilt postoperatively.

### Lenke type 3

Lenke Type 3 is characterized by double major curves. Both the MTC and the TL/LC are structural, whereas the PTC is nonstructural. Further, the Cobb angle of the MTC is greater than that of the TL/LC. According to lumbar spine modifier, it is further categorized into Types 3 A, 3B, and 3 C, with Type 3 C being the most prevalent. For this type, a posterior surgical approach can be employed to achieve fusion of both the MTC and TL/LC. UIV selection follows the same principles as in Lenke Type 1, considering the Cobb angle and PTC flexibility, as well as shoulder balance. However, LIV selection remains a subject of debate, with options including L2, L3, or L4.

#### Non-selective fusion

Lenke et al. [1] indicated that both the MTC and the TL/LC in Lenke Type 3 are structural curves, requiring a double major curve fusion. However, STF can be considered in cases of Lenke type 3 C where the Cobb angle, AVT, and AVR of the MTC are significantly greater than those of the TL/LC. Behensky et al. [34] conducted a retrospective study involving 36 patients with Lenke Type 3 C who underwent STF and revealed that 10 (28%) patients demonstrated postoperative coronal imbalance (> 2 cm). In a case-control study by Singla et al. [35], 74 patients with Lenke Type 3 were categorized into STF and non-STF groups. They revealed that the non-STF group demonstrated a lower incidence of coronal imbalance, higher lumbar curve correction rates, and better correction of the lumbar apical translation. Consequently, they indicated that the trade-off of reduced fused segments at the expense of postoperative coronal balance warrants further consideration. Chang et al. [36] proposed indications for applying the Guan–Din technique for STF in Lenke Type 3 C and 4 C, including: (1) lumbar side bending Cobb angle less than 45°; (2) thoracolumbar kyphosis (T10–L2) less than 20°. Based on these criteria, patients with Lenke Type 3 are recommended to undergo double major curve fusion. However, STF for patients with Lenke Type 3 C should be approached with extreme caution, as it may cause postoperative decompensation of the TL/LC.

#### Selection of LIV

Lenke types 3 and 6 are characterized by structural MTC and TL/LC, thereby adhering to similar principles for LIV selection. Lenke et al. [1] proposed the following criteria for LIV selection for patients undergoing non-selective fusion: (1) When selecting L3 as LIV, the apical vertebra should be the L1/2 disc or cephalad, the L3/4 disc on the convex side of the TL/LC should be neutral or closed, and the vertebral rotation grade of L3 should be 1.5 or less according to the Nash–Moe classification; (2) When selecting L4, the apex vertebra should be L2 or caudad, the L3/4 disc on the convex side of the TL/LC should be open, and the vertebral rotation of L4 should be grade Ⅰ Nash–Moe rotation or greater. Wang et al. [37] retrospectively analyzed 25 patients with Lenke Types 3 C and 6 C who underwent posterior double major curve fusion, with LIV selected at L2, L3, or L4. After a 2-year follow-up, all patients demonstrated satisfactory corrective outcomes, with low postoperative adding-on and trunk shift incidences. In terms of clinical functional scores, Duramaz et al. [38] retrospectively analyzed 90 patients with Lenke Type 3 C, categorizing them into three groups based on LIV selection at L2, L3, and L4. Their results revealed that patients in the L3 group achieved the best Scoliosis Research Society questionnaires-22 (SRS-22)scores. Further, the results of Oswestry Disability Index (ODI) revealed that the L3 group demonstrated a higher of minimal disability incidence, whereas the L2 group exhibited a higher moderate disability rate and the L4 group displayed a greater severe disability prevalence.

However, some scholars advocate for selecting the LIV at or above the LEV of the TL/LC [39]. Wang et al. [40] investigated the correlation between LIV and the lumbar apical vertebra (LAV), encompassing 40 patients with Types 3 C and 6 C spinal deformities. Based on the association between LIV and LAV, the participants were categorized into three groups: group A (LIV above LAV, *n* = 11), group B (LIV at LAV, *n* = 11), and group C (LIV below LAV, *n* = 18). After a two-year follow-up period, no statistically significant differences were found among the three groups concerning scoliosis correction rates and coronal balance. Consequently, they recommend selecting the LAV as the LIV, as this approach not only achieves satisfactory deformity correction but also preserves a greater number of mobile spinal segments. Notably, Chilakapati et al. [41] revealed that overall lumbar lordosis increased due to an increased lordosis in the instrumented segments and a smaller decrease in lordosis below the LIV when performing double major curve fusion. Therefore, they recommended that surgeons should be cautious of the tendency to develop instrumented lumbar lordosis with a compensatory loss of lordosis below LIV which potentially results in poor long-term outcomes in adulthood.

### Lenke type 4

Lenke Type 4 is characterized by three structural curves, with the MTC and TL/LC being major curves, and the PTC as a secondary curve. Based on lumbar spine modifier, it is further classified into Types 4 A, 4B, and 4 C. Representing approximately 3% of cases [3], Lenke Type 4 is the least prevalent category within the Lenke classification system, causing a relative scarcity of surgical studies addressing this subtype. Posterior surgical approaches are typically employed for Lenke type 4 to achieve fusion of all three curves. The selection criteria for the UIV adhere to the same principles applied in Lenke Type 2, with particular consideration of overall shoulder balance. Similarly, the criteria for selecting the LIV are consistent with those established for Lenke Type 3.

### Lenke type 5

Lenke Type 5 is characteristic with structural TL/LC and nonstructural PTC and MTC. According to the modified lumbar curve classification, only Type 5 C is included. Surgical intervention for this type can be approached through either an anterior or posterior approach for selective thoracolumbar/lumbar fusion (SLF). UIV and LIV selection varies based on the surgical approach. UIV can be selected as the upper end vertebra (UEV) for anterior surgeries, whereas UIV selection in posterior surgeries remains a subject of debate, with options including either the UEV or the vertebra above the UEV. Similarly, the LIV can be selected as the LEV for anterior surgeries, whereas LIV selection in posterior surgeries is also contentious, with choices including either the LEV or the vertebra below the LEV.

#### Selection of surgical approach

Lenke et al. [1] indicated that patients with Lenke Type 5 C could opt for SLF via either an anterior or posterior surgical approach. A four-year follow-up comparative study conducted by Dong et al. [42] in 2015 revealed no significant differences between the ASF and PSF groups regarding the correction rates of TL/LC and the spontaneous correction rates of the thoracic curve. Conversely, Zhang et al. [43] retrospectively analyzed data from 45 patients with Lenke Type 5 C who underwent PSF, revealing that PSF effectively achieved spontaneous correction of the thoracic curve while maintaining coronal and sagittal balance. However, patients with moderate Lenke Type 5 C were recommended maximal correction to avoid undercorrection. A meta-analysis conducted by Liang et al. [44] compared the surgical outcomes of ASF and PSF, revealing no significant differences in the changes of TL/LC and MT Cobb angles, as well as overall trunk balance in the long-term correction between the two approaches. Nonetheless, each method presented distinct advantages and disadvantages: ASF involved fewer levels of spinal fusion, whereas PSF facilitated greater thoracic kyphosis and lumbar lordosis. Consequently, the authors suggested that the selection between anterior and posterior approaches should depend on a comprehensive assessment of patient-specific factors and the surgeon’s technical expertise. However, surgeons prefer PSF in clinical practice, because ASF requires an abdominal approach, which increases the surgical complexity and associated risks.

#### Selection of UIV

Lenke et al. [1] developed criteria for UIV selection, advocating for selecting UEV as the UIV in anterior approaches and the one or two vertebrae above the UEV in posterior approaches. However, several researchers have aimed to lower the segments selected for the UIV in both anterior and posterior surgical approaches. In particular, Sudo et al. [45] compared the clinical outcomes of two groups of patients in anterior surgery: one group with UEV as the UIV (14 cases) and the other with the vertebra below the UEV (16 cases). Their results indicated a significantly lower correction rate of the major curve in the group that used the vertebra below the UEV (74% versus 86%). Similarly, Okada et al. [46] conducted a comparison with two groups: one using UEV (10 cases) and the other utilizing the vertebra below the UEV (19 cases), and they similarly observed a marked reduction in the correction rate of the major curve for the latter group (72% versus 86%). However, they demonstrated no significant differences in SRS-22 scores or radiographic parameters, such as coronal and sagittal balance, between the two groups.

In summary, reducing the segments selected for UIV may diminish the correction rate of the major curve, thereby requiring careful consideration by the surgeons when making decisions. In 2020, Qiu Yong et al. [47, 48] introduced the principle of hyper-selective fusion for UIV in posterior surgeries, indicating that when the Risser sign exceeds grade 2 and the compensatory thoracic curve over 15°, the vertebra just below the UEV can be selected as the UIV to prevent decompensation of the thoracic curve. Subsequently, his research team (Gu et al.) [49] proposed the modified S-line, which was the line connecting the centers of the concave-side pedicles of the UEV and LEV at baseline. Modified S-line+ (UEV being to the right of the LEV) is a modified S-line tilt to the right. Their study revealed that the modified S-line could predict postoperative coronal decompensation, and the vertebra just below the UEV was recommended as UIV with a modified S-line + status and a thoracic compensatory curve. Further, surgeons should be cautious to prevent selecting the UIV within the thoracolumbar region to minimize the incidence of proximal junctional kyphosis postoperatively [50].

#### Selection of LIV

Lenke et al. [1] proposed selection criteria for the LIV, recommending the LEV as the LIV for anterior approaches and either the LEV or the vertebra just below the LEV for posterior approaches. Some studies have investigated the selection of the vertebra above the LEV in anterior surgeries, revealing a reduction in the correction rate of the major curve and an increased distal adding-on incidence [51, 52]. Sun et al. [53] conducted a comparative study assessing the efficacy of selecting either the LEV or the vertebra just below the LEV as the LIV in posterior surgeries. Their results indicated no significant differences in clinical and radiographic parameters between fusing to the vertebra below the LEV and fusing to the LEV in patients with moderate TL/LC (Cobb angle ranging from 30° to 60°). Therefore, they recommend fusing to the LEV in such cases. Li et al. [54] indicated that when the rotation of the LEV exceeds grade 2 or the distance from the LEV to the CSVL is substantial, the LIV for posterior surgery should be selected as the vertebra below the LEV. Yang et al. [55] determined specific scenarios in which the vertebra below the LEV should be selected as the LIV: (1) the Cobb angle of the TL/LC exceeds 60°; (2) the disc angle between the LEV and the vertebra below cannot reach 0° on bending radiographs; (3) the LEV exhibits significant tilting or displacement. Besides, Zhuang et al. [56] proposed selection principles for the LIV after following up with 138 patients: (1) the LIV should be the most cranial vertebra intersecting with the vertical line of the bilateral iliac crests (King–CSVL); (2) Nash–Moe rotation is grade I or less on the standing anteroposterior radiograph; (3) King–CSVL intersects between the LIV pedicles on the concave bending film; (4) the vertebra is not an apical vertebra of a kyphotic curve. Their study revealed that the application of these criteria achieved satisfactory coronal plane correction and trunk balance without increasing the number of fused segments. Subsequently, their research team (Li et al.) [57, 58] reported comparable correction in coronal and sagittal planes between the L3 and L4 groups, and terminating the distal fusion level at L3 was practical for patients with mild to moderate Lenke Type 5 C. However, Li et al. [59] revealed that LIV was best set at L4, which facilitated thoracic kyphosis recovery, symptom improvement, and complication and pelvic deformity prevention. Shao et al. proposed that combining LIV de-rotation technique with posterior PSF can serve as an optimized strategy for Lenke type 5/6 curves, particularly for high-risk patients with preoperative LIV tilt > 20° or LIV disc angle > 5°, in order to reduce distal compensatory load [60].

### Lenke type 6

Lenke Type 6 is characterized by a major TL/LC and a structural minor MTC with a nonstructural PTC. According to the lumber spine modifier, only Type 6 C is included. This type is suitable for double curve fusion of MTC and TL/LC. The selection criteria for the UIV are the same as those for Lenke Type 1, considering the Cobb angle and PTC flexibility, as well as shoulder balance. The selection criteria for the LIV are the same as those for Lenke Type 3 C.

Lenke et al. [1] indicated that both the MTC and the TL/LC in Lenke Type 6 C are structural curves that require double curve fusion. However, when the Cobb angle, AVT, and AVR of the TL/LC are significantly greater than those of the MTC, anterior or posterior SLF can be considered. A case-control study conducted by Chang et al. [61] in 2021 compared the postoperative correction effects of SLF between patients with Lenke Type 5 C and Lenke Type 6 C. The study revealed that patients with Lenke Type 6 C demonstrated significant spontaneous correction of the MTC after SLF and achieved SRS-22 scores similar to those of Lenke Type 5 C at final follow-up. Therefore, they concluded that SLF is a viable treatment option for patients with Lenke Type 6 C.

## Discussion

**Fig. 2:**
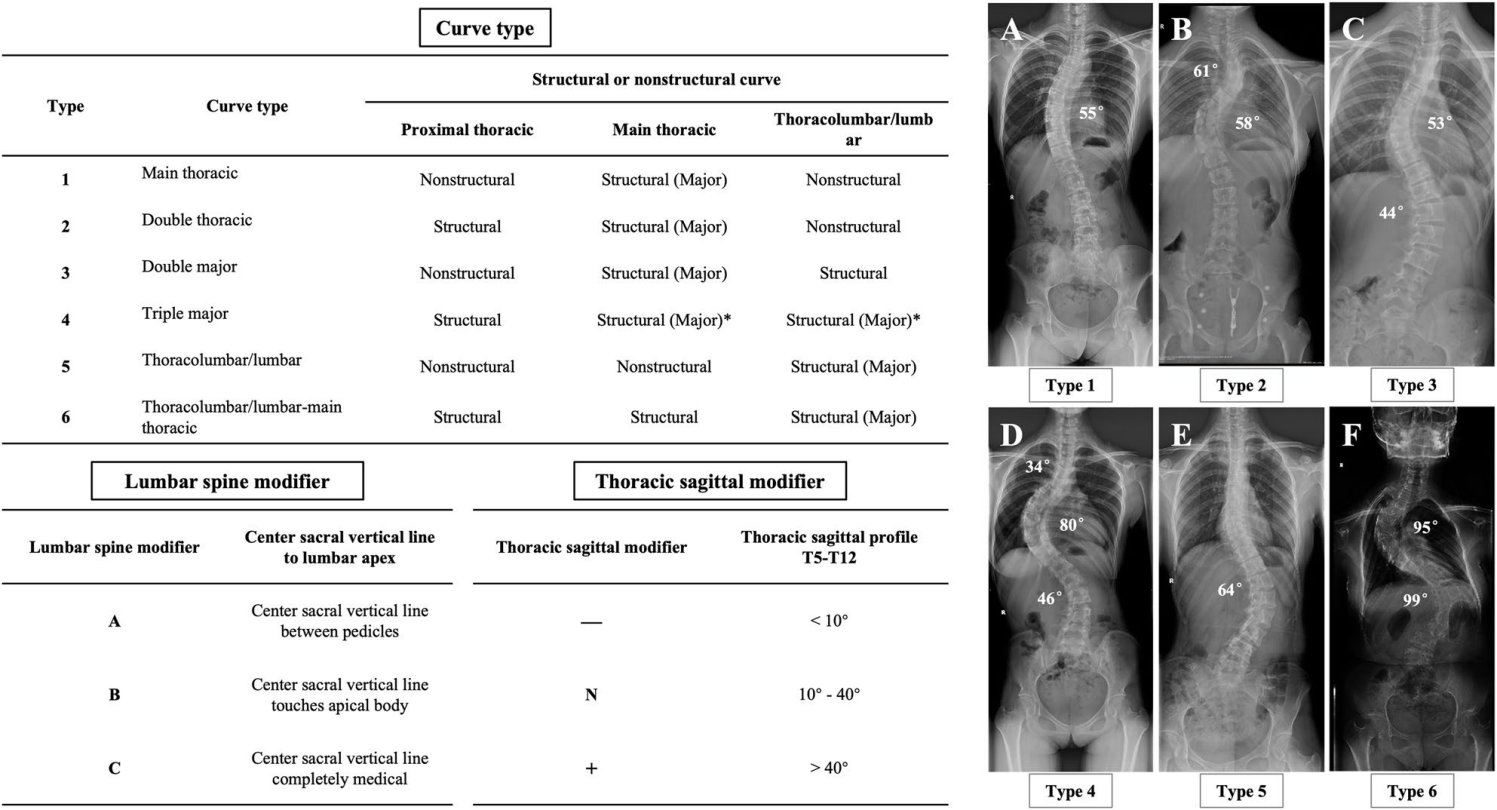
Chart showing the Lenke classification system. * indicates that either the main thoracic or thoracolumbar/lumbar curve may be the major curve in Lenke Type 4

In summary, Lenke et al. [1, 3] proposed a surgical guideline for AIS based on the Lenke classification system in 2001, including selective fusion, surgical approach, and UIV and LIV selection, which provided significant guidance for the surgical treatment of AIS. This guideline has been continuously refined and developed over more than a decade of clinical application by several scholars, including Lenke himself [39, 62]. For instance, Lenke et al. modified LIV selection for Lenke Type 1 and 2 from SV to TV in 2020, which not only achieved excellent clinical outcomes but reduced the number of fused segments. Interestingly, we revealed that recent studies have primarily focused on validating the fusion strategies proposed by Lenke in earlier years. Although some innovative surgical strategies have been proposed, groundbreaking studies that challenge the fusion strategies based on the Lenke classification have not been reported. Certainly, several points of contention remain regarding the surgical strategies for AIS, such as the feasibility of STF for Lenke Type 1 C and 2 C, the selection of UIV for Lenke Type 2, and the selection of LIV for Lenke Type 3 and 5 C. Therefore, we summarized the overall surgical strategies for AIS by integrating Lenke’s proposed surgical guidelines and the latest literature (Table 1**)**. The quality of and level of evidence for the included studies were shown in Supplementary File 1. Furthermore, we created a chart that summarized the Lenke classification system in a clearer and more understandable way (Fig. 2).

**Table 1:** Fusion strategies for AIS based on Lenke classification system

Lenke classification	Surgical Approach	Fusion Range	Selection of UIV	Selection of LIV
Type 1	Posterior approach (first choice)	1 A and 1B: STF 1 C: conditions for STF: (1) MTC > TL/LC, with TL/LC < 25° and/or T10-L2 kyphosis angle ≥ 20°on side bending; (2) the ratio criteria of MTC: TL/LC Cobb magnitude, AVT, and AVR ≥ 1.2	Left shoulder is higher: UIV = T2 Shoulders are balanced: UIV = T3 or T4 Right shoulder is high: UIV = T4 or T5	Posterior approach: LIV = TV
	Anterior approach (thoracoscopy)			Anterior approach: LIV = LEV
Type 2	Posterior approach	2 A and 2B: selective double thoracic curves fusion	UIV = T1 or T2	Same to Type 1
		2 C: the conditions of selective double thoracic curves fusion are same to Type 1 C		
Type 3	Posterior approach	Double major curves fusion	Same to Type 1	LIV = L3 or L4
Type 4	Posterior approach	Triple major curves fusion	Same to Type 2	Same to Type 3
Type 5	Posterior approach (first choice)	Selective thoracolumbar/lumbar fusion	Posterior approach: UIV = UEV or (UEV-1)	Posterior approach: LIV = LEV or (LEV + 1)
	Anterior approach		Anterior approach: UIV = UEV	Anterior approach: LIV = LEV
Type 6	Posterior approach	Double major curves fusion	Same to Type 1	Same to Type 3

*AIS* adolescent idiopathic scoliosis, *UIV* upper instrumented vertebra, *LIV* lower instrumented vertebra, *STF* selective thoracic fusion, *MTC* main thoracic curve, *TL/LC* thoracolumbar/lumbar curve, *T* thoracic vertebra, *L* lumbar vertebra, *AVT* apical vertebral translation, *AVR* apical vertebral rotation, *TV* touching vertebra, *LEV* lower end vertebra, *UEV* upper end vertebra

It is clinically significant to conduct multicenter, large-sample case studies to validate and optimize surgical strategies based on the current Lenke classification system for AIS, especially certain innovative surgical strategies, such as the MSLV concept proposed by Munakata et al. [18] and the LSTV-1 rule proposed by Qin et al. [26]. Furthermore, surgical strategies need to be constantly updated considering the continuous development of orthopedic internal fixation instruments and new technologies, such as navigation, robotic spine surgery, and 3D printing. For instance, the Guan–Din technique proposed by Chang et al. [40] has enabled the indications for STF to expand gradually by relying on a powerful pedicle internal fixation system.

The Lenke classification system remains a two-dimensional classification method that cannot reflect the rotational degree of the vertebra. Therefore, incorporating parameters, such as vertebral rotation into the surgical strategies for AIS (for example, avoiding the selection of significantly rotated vertebrae as the LIV), is expected to further improve the scientific rigor and precision of surgical decision-making [63, 64]. In addition, the Scoliosis Research Society (SRS), Aubin and Lenke proposed the SRS–Lenke–Aubin 3D classification, which incorporates the orientation of the regional plane of deformation and the apical vertebral rotation within the classical classification system [65]. The novel 3D spinal morphological classification provides a new reference for personalized diagnosis and treatment, yet its efficacy requires further validation in clinical practice and multicenter validation [66]. Moreover, emerging robotic technologies have demonstrated substantial potential in improving the accuracy and safety of AIS surgery [67]. Previous studies have shown that robot-assisted procedures may reduce the need for extending fusion levels due to difficulties in pedicle screw insertion and minimizing the use of laminar hooks [68]. With continued advancements in robotic systems and their potential integration with 3D classification frameworks, surgical strategy selection may be further optimized in the future. Finally, screw density is also a critical factor in surgical decision-making. Optimizing screw density can reduce intraoperative blood loss, postoperative pain, and surgical costs without compromising correction efficacy. However, the optimal screw density strategy still requires validation through further clinical trials [69, 70].

This review has several limitations. First, its focus was confined to the advances in fusion level selection and surgical approaches for AIS based on the Lenke classification system, and therefore did not encompass other important surgical aspects such as surgical efficiency, surgical safety, cost-effectiveness, postoperative pain relief, or early discharge. Moreover, this review did not address non-fusion techniques, particularly vertebral body tethering (VBT), which represents an emerging motion-preserving alternative for selected patients. Future studies should integrate these dimensions to provide a more comprehensive understanding of surgical decision-making and outcomes in the management of AIS.

## Conclusion

Fusion surgery guidelines based on the Lenke classification system remain the current standard for treating AIS. In recent years, several scholars have refined and expanded upon this basis, achieving satisfactory results, such as the orthopedic rate improvement and fewer postoperative complications. However, some details remain controversial. Considering the development of surgery and related technologies, surgical strategies need to be further optimized and 3D classification systems should be developed.

## Supplementary Information

Supplementary Material 1.

## Data Availability

No datasets were generated or analysed during the current study.

## Abbreviations

3D: three-dimensional
AIS: adolescent idiopathic scoliosis
ASF: anterior spinal fusion
AVR: apical vertebral rotation
AVT: apical vertebral translation
CSVL: central sacral vertical line
LAV: lumbar apical vertebra
LEV: lower end vertebra
LIV: lower instrumented vertebra
MSL: modified Shinshu line
MSLV: modified Shinshu line vertebra
MTC: main thoracic curve
nSTV: non-substantially touched vertebra
NV: neutral vertebra
ODI: Oswestry Disability Index
PSF: posterior spinal fusion
PTC: proximal thoracic curve
SLF: selective thoracolumbar/lumbar fusion
SRS: Scoliosis Research Society
SRS-22: Scoliosis Research Society questionnaires-22
STF: selective thoracic fusion
STV: substantially touched vertebra
SV: stable vertebra
SV-1: 1 vertebra below the stable vertebra
SV-2: 2 vertebrae below the stable vertebra
TL/ LC: thoracolumbar/lumbar curve
TV: touching vertebra
UEV: upper end vertebra
UIV: upper instrumented vertebra
