# Supplementary material for: Advances in fusion level selection and surgical approaches for adolescent idiopathic scoliosis based on the Lenke classification system: a narrative review
Source: BMC Surg. 2026 Jan 5;26:102. doi: 10.1186/s12893-025-03481-9 (PMC12870182; doi:10.1186/s12893-025-03481-9)
Supplement: Supplementary file 1 — Supplementary Material 1. [file 12893_2025_3481_MOESM1_ESM.docx]

**Supplementary File 1.** The characteristics of the studies included in the review

The quality of original articles included in the review was assessed by the Risk of Bias in Nonrandomized Studies of Interventions (ROBINS-I) [1]. Risk of biases in 7 domains were evaluated in terms of confounding, selection of participants, classification of interventions, deviations from intended interventions, missing data, measurement of outcomes, and selection of the reported result. The judgment of risk of bias was classified as low, moderate, serious, and critical risk of bias. The level of evidence of original articles included in the review was assessed by the Grading of Recommendations Assessment, Development, and Evaluation (GRADE) criteria [2]. Based on these evaluations, the level of evidence was classified as high, moderate, low, or very low. The overall confidence level of the included systematic reviews included in the review was rated by AMSTAR 2 and was classified as high, moderate, low, critically low confidence [3].

**Table 1.** The quality and level of evidence of original articles included in the review

| Author | Publication year | Study design | Patients included (n) | Quality assessment | Level of evidence |
| --- | --- | --- | --- | --- | --- |
| Lenke LG, et al. [2] | 2001 | Retrospective | 315 | Moderate | Low |
| Newton PO, et al. [4] | 2003 | Retrospective | 203 | Moderate | Low |
| Crawford CH, et al. [5] | 2013 | Retrospective | 264 | Serious | Low |
| Wang Y, et al. [7] | 2012 | Retrospective | 29 | Serious | Low |
| Kwan MK, et al. [8] | 2018 | Retrospective | 44 | Serious | Low |
| Lenke LG, et al. [9] | 2003 | Retrospective | 65 | Critical | Low |
| Kaya O, et al. [10] | 2022 | Retrospective | 55 | Serious | Low |
| Potter BK, et al. [11] | 2005 | Retrospective | 20 | Serious | Moderate |
| Newton PO, et al. [12] | 2013 | Prospective | 136 | Moderate | Low |
| Gu H, et al. [13] | 2022 | Retrospective | 45 | Serious | Very low |
| Levin R, et al. [14] | 2005 | Retrospective | 45 | Serious | Low |
| Zhang HQ, et al. [15] | 2012 | Prospective | 64 | Moderate | Moderate |
| Chan CYW, et al. [16] | 2016 | Prospective | 100 | Serious | Moderate |
| Brooks JT, et al. [17] | 2018 | Prospective | 626 | Serious | Low |
| Munakata R, et al. [18] | 2020 | Retrospective | 45 | Serious | Low |
| Oba H, et al. [19] | 2019 | Retrospective | 48 | Moderate | Low |
| Sielatycki JA, et al. [20] | 2019 | Retrospective | 145 | Serious | Moderate |
| Hiett A, et al. [21] | 2020 | Retrospective | 132 | Serious | Moderate |
| Suk SI, et al. [22] | 2003 | Retrospective | 42 | Moderate | Low |
| Beauchamp EC, et al. [23] | 2020 | Retrospective | 299 | Moderate | Very Low |
| Qin X, et al. [26] | 2022 | Retrospective | 94 | Moderate | Very Low |
| Andre E, et al. [27] | 2023 | Retrospective | 105 | Moderate | Very Low |
| Guo W, et al. [29] | 2025 | Retrospective | 213 | Moderate | Low |
| Berlin C, et al. [30] | 2023 | Retrospective | 127 | Serious | Moderate |
| Li M, et al. [31] | 2009 | Retrospective | 25 | Serious | Low |
| Jiang J, et al. [32] | 2017 | Retrospective | 30 | Serious | Low |
| Mimura T, et al. [33] | 2022 | Retrospective | 55 | Moderate | Very Low |
| Behensky H, et al. [34] | 2007 | Retrospective | 36 | Serious | Very Low |
| Singla A, et al. [35] | 2014 | Retrospective | 74 | Serious | Very Low |
| Chang KW, et al. [36] | 2014 | Retrospective | 320 | Serious | Moderate |
| Wang Y, et al. [37] | 2012 | Retrospective | 25 | Serious | Low |
| Duramaz A, et al. [38] | 2020 | Retrospective | 90 | Serious | Low |
| Wang Y, et al. [40] | 2012 | Retrospective | 40 | Serious | Low |
| Chilakapati S, et al. [41] | 2023 | Retrospective | 77 | Serious | Low |
| Dong Y, et al. [42] | 2016 | Retrospective | 53 | Serious | Low |
| Zhang Y, et al. [43] | 2017 | Retrospective | 45 | Serious | Low |
| Sudo H, et al. [45] | 2016 | Retrospective | 30 | Serious | Low |
| Okada E, et al. [46] | 2015 | Retrospective | 29 | Serious | Very Low |
| Shu S, et al. [47] | 2020 | Prospective | 80 | Serious | Low |
| Bao HD, et al. [48] | 2021 | Prospective | 28 | Moderate | Low |
| Gu Q, et al. [49] | 2024 | Retrospective | 92 | Moderate | Low |
| Li QD, et al. [50] | 2023 | Retrospective | 98 | Moderate | Low |
| Sudo H, et al. [51] | 2016 | Retrospective | 30 | Moderate | Low |
| Dubory A, et al. [52] | 2017 | Retrospective | 42 | Moderate | Low |
| Sun Z, et al. [53] | 2014 | Retrospective | 37 | Moderate | Low |
| Li J, et al. [54] | 2011 | Retrospective | 27 | Serious | Very Low |
| Yang X, et al. [55] | 2018 | Retrospective | 56 | Serious | Very Low |
| Zhuang Q, et al. [56] | 2021 | Retrospective | 138 | Moderate | Very Low |
| Li C, et al. [57] | 2025 | Retrospective | 106 | Moderate | Low |
| Li Z, et al. [58] | 2023 | Retrospective | 82 | Moderate | Low |
| Li J, et al. [59] | 2023 | Retrospective | 94 | Serious | Low |
| Shao X, et al. [60] | 2022 | Retrospective | 53 | Serious | Low |
| Chang SY, et al. [61] | 2021 | Retrospective | 31 | Serious | Low |
| He Z, et al. [63] | 2020 | Retrospective | 196 | Moderate | Low |
| Sarwahi V, et al. [64] | 2022 | Retrospective | 416 | Moderate | Low |
| Pasha S, et al. [66] | 2018 | Retrospective | 67 | Moderate | Low |
| Ma B, et al. [68] | 2025 | Retrospective | 47 | Moderate | Low |
| Lu Z, et al. [70] | 2025 | Retrospective | 213 | Serious | Low |

**Table 2.** The overall confidence level of the included systematic reviews included in the review

| Author | Publication year | Overall confidence |
| --- | --- | --- |
| Boniello AJ, et al. [6] | 2015 | Critically Low |
| Ifthekar S, et al. [24] | 2023 | Critically low |
| Liu CW, et al. [25] | 2020 | Critically low |
| Yang M, et al. [28] | 2018 | Critically low |
| Liang W, et al. [44] | 2023 | Critically low |
| Cui W, et al. [67] | 2024 | Critically low |
| Zheng B, et al. [69] | 2025 | Low |

**References**

1. The Risk Of Bias In Non-randomized Studies – of Interventions, Version 2 (ROBINS-I V2) assessment tool. 2024. https://www.riskofbias.info/welcome/robins-i-v2. Accessed 30 Aug 2025.

2. Guyatt GH, Oxman AD, Vist GE, Kunz R, et al. GRADE: an emerging consensus on rating quality of evidence and strength of recommendations. BMJ. 2008;336(7650):924-6. https://doi.org/10.1136/bmj.39489.470347.AD

3. Shea BJ, Reeves BC, Wells G, et al. AMSTAR 2: a critical appraisal tool for systematic reviews that include randomised or non-randomised studies of healthcare interventions, or both. BMJ. 2017;358:j4008. https://doi.org/10.1136/bmj.j4008
